# Genome Sequence of the Acrystalliferous *Bacillus thuringiensis* Serovar Israelensis Strain 4Q7, Widely Used as a Recombination Host

**DOI:** 10.1128/genomeA.00231-14

**Published:** 2014-04-03

**Authors:** Haeyoung Jeong, Seung-Hwan Park, Soo-Keun Choi

**Affiliations:** a Korean Bioinformation Center, Korea Research Institute of Bioscience and Biotechnology (KRIBB), Daejeon, Republic of Korea; b Super-Bacteria Research Center, KRIBB, Daejeon, Republic of Korea

## Abstract

*Bacillus thuringiensis* serovar israelensis is well known for its mosquitocidal activity and has long been used as a biopesticide. Herein, we present the genome sequence of *B. thuringiensis* serovar israelensis strain 4Q7, a plasmid-cured derivative with higher transformation efficiency than wild types.

## GENOME ANNOUNCEMENT

*Bacillus thuringiensis* is a ubiquitous, spore-forming, Gram-positive bacterium that produces a parasporal protein crystal (generally encoded by plasmids) during sporulation (1). Because of the insecticidal activity of its crystals, which is specific only to a single group or species of insect, *B. thuringiensis* has long been used as a safe and ecofriendly biological pesticide to protect crops against harmful insects (2). Specifically, *B. thuringiensis* serovar israelensis, first discovered in 1976, has been used worldwide in large-scale programs for controlling mosquitoes (3).

*Bacillus thuringiensis*, *Bacillus cereus* (*sensu stricto*), and *Bacillus anthracis*, collectively called the *B. cereus* group, form a highly homogeneous subdivision of the genus *Bacillus* and are often regarded as a single species owing to their high genetic relatedness (4, 5). Among the species in the *B. cereus* group, *B. thuringiensis*, the only species that has been recognized as being safe to humans and animals, has been subject to research for improving the efficacy of *B. thuringiensis*-based biopesticides. Because most *B. thuringiensis* strains harbor a number of diverse plasmids, a plasmidless strain may serve as an indispensable tool for the genetic engineering of *B. thuringiensis* and as a surrogate host for other members of the *B. cereus* group. *B. thuringiensis* serovar israelensis 4Q7, available through the Bacillus Genetic Stock Center (Columbus, OH), is an acrystalliferous, plasmidless *B. thuringiensis* strain (6) widely used as a recombination host.

The 4Q7 genome was sequenced using an Illumina HiSeq 2000 system at the National Instrumentation Center for Environmental Management (Seoul, Korea). A total of 41,404,492 paired-end reads (101 nucleotides [nt] and 3.17 Gb in total) were produced from an ~430-bp genomic library and were preprocessed and *de novo* assembled using the CLC Genomics Workbench version 6.5.1. The final assembly, which takes >24× read scaffolds into account (334× at minimum), consists of 5,042,766 bases in 44 scaffolds (1,676 Ns) with a G+C content of 35.3%, which can be broken into 55 contigs. The maximum scaffold length and *N*_50_ were 607,569 bp and 287,111 bp, respectively. Automatic genome annotation, performed using the RAST server (7), predicted 115 RNA genes and 5,193 coding sequences, 41% of which were categorized into subsystems. As expected, no *Bt* toxin genes were identified when all translated coding sequences (CDSs) were subjected to the BtToxinScanner (8).

An average nucleotide identity analysis using JSpecies software (9) revealed that, among the completely sequenced strains in the *B. cereus* group, *B. thuringiensis* HD-789 (99.9%) and *B. cereus* G9842 (99.3%) are the most similar to 4Q7. We found that contig 5 (235 kb), encoding mostly hypothetical proteins and phage-related proteins, is specific to 4Q7, while the ~490-kb region common to other *B. thuringiensis* genomes (bp 1783089 to 2274756 in the HD-798 strain, for example) is missing in 4Q7. We believe these genomic differences account for the phenotypic characteristics of 4Q7.

### Nucleotide sequence accession numbers.

This whole-genome shotgun project has been deposited at DDBJ/EMBL/GenBank under the accession number JEOC00000000. The version described in this paper is version JEOC01000000.

## References

[1] Schnepf E Crickmore N Van Rie J Lereclus D Baum J Feitelson J Zeigler DR Dean DH 1998 *Bacillus thuringiensis* and its pesticidal crystal proteins. Microbiol. Mol. Biol. Rev. 62:775–806 9729609 10.1128/mmbr.62.3.775-806.1998 PMC98934

[2] Sanahuja G Banakar R Twyman RM Capell T Christou P 2011 *Bacillus thuringiensis*: a century of research, development and commercial applications. Plant Biotechnol. J. 9:283–300. 10.1111/j.1467-7652.2011.00595.x 21375687

[3] Goldberg LH Margalit J 1977 A bacterial spore demonstrating rapid larvicidal activity against *Anopheles sergentii*, *Uranotaenia unguiculata*, *Culex inivitattos*, *Aedes aegypti* and *Culex pipiens*. Mosq. News 37:355–358

[4] Rasko DA Altherr MR Han CS Ravel J 2005 Genomics of the *Bacillus cereus* group of organisms. FEMS Microbiol. Rev. 29:303–329. 10.1016/j.fmrre.2004.12.005 15808746

[5] Vilas-Bôas GT Peruca AP Arantes OM 2007 Biology and taxonomy of *Bacillus cereus*, *Bacillus anthracis*, and *Bacillus thuringiensis*. Can. J. Microbiol. 53:673–687. 10.1139/W07-029 17668027

[6] Clark BD 1987 Characterization of plasmids from *Bacillus thuringiensis* var. *israelensis*. Ph.D. thesis Ohio State University, Columbus, OH

[7] Aziz RK Bartels D Best AA DeJongh M Disz T Edwards RA Formsma K Gerdes S Glass EM Kubal M Meyer F Olsen GJ Olson R Osterman AL Overbeek RA McNeil LK Paarmann D Paczian T Parrello B Pusch GD Reich C Stevens R Vassieva O Vonstein V Wilke A Zagnitko O 2008 The RAST server: rapid annotations using subsystems technology. BMC Genomics 9:75. 10.1186/1471-2164-9-75 18261238 PMC2265698

[8] Ye W Zhu L Liu Y Crickmore N Peng D Ruan L Sun M 2012 Mining new crystal protein genes from *Bacillus thuringiensis* on the basis of mixed plasmid-enriched genome sequencing and a computational pipeline. Appl. Environ. Microbiol. 78:4795–4801. 10.1128/AEM.00340-12 22544259 PMC3416374

[9] Richter M Rosselló-Móra R 2009 Shifting the genomic gold standard for the prokaryotic species definition. Proc. Natl. Acad. Sci. U. S. A. 106:19126–19131. 10.1073/pnas.0906412106 19855009 PMC2776425

